# On the quasi-isometric and bi-Lipschitz classification of 3D Riemannian Lie groups

**DOI:** 10.1007/s10711-020-00532-8

**Published:** 2020-04-28

**Authors:** Katrin Fässler, Enrico Le Donne

**Affiliations:** 1 Department of Mathematics, University of Fribourg, Fribourg, Switzerland; 2 Present Address: Department of Mathematics and Statistics, University of Jyväskylä, Jyväskylä, Finland

**Keywords:** Lie groups, Quasi-isometric, Bi-Lipschitz, Isometric, Riemannian manifold, Classification, 53C23, 22D05, 20F65

## Abstract

This note is concerned with the geometric classification of connected Lie groups of dimension three or less, endowed with left-invariant Riemannian metrics. On the one hand, assembling results from the literature, we give a review of the complete classification of such groups up to quasi-isometries and we compare the quasi-isometric classification with the bi-Lipschitz classification. On the other hand, we study the problem whether two quasi-isometrically equivalent Lie groups may be made isometric if equipped with suitable left-invariant Riemannian metrics. We show that this is the case for three-dimensional simply connected groups, but it is not true in general for multiply connected groups. The counterexample also demonstrates that ‘may be made isometric’ is not a transitive relation.

## Introduction

### List of groups of dimension at most three

Following the Bianchi classification (see e.g. Theorems 1.4 and 1.5 in [12, Chapter 7]), we start by listing the connected real Lie groups of dimension at most three:

- **Lie groups of dimension one** $\mathbb {R}$, $\mathbb {T}^1$.
- **Lie groups of dimension two** $\mathbb {R}^2$, $\mathbb {R}\times \mathbb {T}^1$, $\mathbb {T}^2$, ${\text {Aff}}^{+}(\mathbb {R})$.
- **Lie groups of dimension three** $\mathbb {R}^3$, $\mathbb {R}^2 \times \mathbb {T}^1$, $\mathbb {R}\times \mathbb {T}^2$, $\mathbb {T}^3$, $\mathrm {N}_3(\mathbb {R})$, $\mathrm {N}_3^{*}(\mathbb {R})$, $\mathrm {SU}(2)$, $\mathrm {SO}(3)$, $\widetilde{\mathrm {SL}}(2)$, $\{\mathrm {PSL}(2)_k:\; k\in \mathbb {N}\}$, $\widetilde{\mathrm {SE}}(2)$, $\{\mathrm {SE}(2)_k:\; k\in \mathbb {N}\}$, $\mathrm {J}$, $\{\mathrm {D}_{\lambda }:\;0<|\lambda |\le 1\}$, $\{\mathrm {C}_{\lambda }:\;\lambda >0\}$, ${\text {Aff}}^{+}(\mathbb {R})\times \mathbb {R}$, ${\text {Aff}}^{+}(\mathbb {R})\times \mathbb {T}^1$.

Many of these groups are well known: the *k*-dimensional Euclidean group $\mathbb {R}^k$, the *k*-dimensional torus $\mathbb {T}^k=(\mathbb {R}/\mathbb {Z})^k$ and direct products of these groups. Nilpotent but non-Abelian groups are the Heisenberg group $\mathrm {N}_3(\mathbb {R})$ and its quotient $\mathrm {N}_3^{*}(\mathbb {R})$ modulo the group of integer points in the center, when $\mathrm {N}_3(\mathbb {R})$ is seen as upper triangular matrix group. Among the solvable but not nilpotent groups there are ${\text {Aff}}^{+}(\mathbb {R})$ (the group of orientation-preserving affine maps of the real line) and products thereof with $\mathbb {R}$ and $\mathbb {T}^1$, as well as $\widetilde{\mathrm {SE}}(2)$ [the universal cover of the group $\mathrm {SE}(2)$ of orientation preserving isometries of the Euclidean plane] and $\mathrm {SE}(2)_k$ [the *k*-fold cover of $\mathrm {SE}(2)$]. Well-known simple groups are $\mathrm {SU}(2)$ (the special unitary group), $\mathrm {SO}(3)$ (the special orthogonal group), $\widetilde{\mathrm {SL}}(2)$ (the universal cover of the special linear group), and $\mathrm {PSL}(2)_k$ [the *k*-fold cover of the projective special linear group $\mathrm {PSL}(2)$].

Apart from $\widetilde{\mathrm {SL}}(2)$ and $\mathrm {SU}(2)$, all the simply connected groups listed in the previous paragraph are isomorphic to semidirect products $\mathbb {R}^2 \rtimes _A \mathbb {R}$, where $\mathbb {R}$ acts on $\mathbb {R}^2$ by a matrix $A\in \mathrm {Mat}(2\times 2,\mathbb {R})$ such that the Lie group product is given by the following expression:

1.1 $$\begin{aligned} (x,y,z)*_A (x',y',z'):=\left( \begin{pmatrix}x\\ y\end{pmatrix}+ e^{zA}\begin{pmatrix}x'\\ y'\end{pmatrix},z+z'\right) . \end{aligned}$$

One can find a basis $\{E_1,E_2,E_3\}$ for the Lie algebra of $\mathbb {R}^2 \rtimes _A \mathbb {R}$ whose structure constants are given by

1.2 $$\begin{aligned} A= \begin{pmatrix}c_{13}^1&{}\quad c_{23}^1 \\ c_{13}^2&{}\quad c_{23}^2\end{pmatrix}, \end{aligned}$$

and $c_{ij}^k = 0$ for all other cases where $i\le j$ and $k\in \{1,2,3\}$, see for instance [26, §2.2]. The connected 3-dimensional Lie groups which we have not yet introduced are all solvable and also of the form $\mathbb {R}^2 \rtimes _A \mathbb {R}$. For $$A=\left( \begin{array}{cc}1&{}1 \\ 0&{}1\end{array}\right) $$ [respectively $$\left( \begin{array}{cc}1&{}0 \\ 0&{}\lambda \end{array}\right) $$, respectively $$\left( \begin{array}{cc}\lambda &{} 1 \\ -\,1&{} \lambda \end{array}\right) $$], we obtain *J* (respectively $D_{\lambda }$, respectively $C_{\lambda }$).

### Classification results

Standing assumption. *All distances considered are left-invariant Riemannian distances.*

A (not necessarily continuous) map $\Psi :(X,d) \rightarrow (X',d')$ between metric spaces is a *quasi-isometry* if there exist constants $0\le C<\infty$ and $1\le L<\infty$ such that

(i) $$L^{-1}d(x,y)-C\le d'(\Psi (x),\Psi (y))\le L d(x,y) + C$$ for all $$x,y\in X$$,
(ii) for all $x'\in X'$ there is $x\in X$ such that $d'(\Psi (x), {x'})\le C$.

If (i) and (ii) hold with $C=0$, the map $\Psi$ is said to be *bi-Lipschitz*, and if moreover $L=1$, then $\Psi$ is an *isometry*. If *X* and $X'$ are manifolds and if the distances *d* and $d'$ are induced by Riemannian metrics *g* and *g*, respectively, then according to a well-known result by Myers and Steenrod [28], the map $\Psi$ is an isometry exactly if it is a diffeomorphism such that $\Psi ^{*} g' =g$, see also [32, Theorem 5.6.15]. Since any two left-invariant Riemannian distances on a Lie group are bi-Lipschitz equivalent, we can discuss the quasi-isometric and bi-Lipschitz classification of such groups without specifying a metric. On the other hand, the existence of *isometries* between two groups depends on the choice of metrics. As we are interested in the geometric classification of groups, rather than the classification of groups endowed with a specific metric, we study the following property.

#### Definition 1.1

We say that two connected Lie groups *G* and *H* *may be made isometric* if there exist left-invariant Riemannian distances $d_G$ and $d_H$ on *G* and *H*, respectively, such that $(G,d_G)$ and $(H,d_H)$ are isometric.

Definition 1.1 goes back to [5, §1.2], but differs slightly from the original definition, which was formulated for arbitrary topological groups and which required only the existence of left-invariant distances that induce the manifold topology. By [23, Proposition 2.4] isometries between connected Lie groups endowed with such distances are actually isometries for some left-invariant *Riemannian* distances, and hence Definition 1.1 agrees with the definition of [5] in the case of connected Lie groups.

It is easy to show that two Lie groups *G* and *H* may be made isometric if and only if there exists a Riemannian manifold *M* on which both *G* and *H* act simply transitively by isometries, see Proposition 2.1.

If *X* is a fixed model space with a standard distance $d_X$, for instance Euclidean space or the hyperbolic plane, we will also say that “*G* may be made isometric to *X*” if there exists a left-invariant Riemannian distance $d_G$ on *G* such that $(G,d_G)$ and $(X,d_X)$ are isometric.

In Sect. 2, we discuss relations between connected Lie groups of dimension at most three in descending order of strength, that is, we list pairs consisting of groups that

- may be made isometric (Proposition 2.2)
- are bi-Lipschitz (Proposition 2.11)
- are quasi-isometrically homeomorphic (Proposition 2.14)
- are quasi-isometric (Proposition 2.15).

To conclude the quasi-isometric classification given in Theorem 1.2 below, we show that the pairs not appearing in the list (a)–(d) consist of groups that are not quasi-isometrically equivalent.

Classification problems for Lie groups have a long history that dates back to Bianchi’s [2] isomorphic classification of 3-dimensional Lie algebras. This note is concerned with the geometric classification of Lie groups that are additionally equipped with left-invariant Riemannian distances. Gromov [14] in his address to the ICM in 1983 promoted a program to study finitely generated groups with word metrics up to quasi-isometries. This classification problem is related to the quasi-isometric classification of Riemannian manifolds, as the fundamental group of a compact connected Riemannian manifold *M* is a finitely generated group quasi-isometrically equivalent to the universal Riemannian cover $\widetilde{M}$ according to the Švarc-Milnor lemma.

In the first part of this note, we recall the quasi-isometric classification of connected Lie groups up to dimension three. This is the work of several authors who have studied various aspects of the quasi-isometric classification, for instance for solvable groups of a specific form, or under curvature constraints. We list some of these results: Guivarc’h and Jenkins’ [17, 19] characterization of connected Lie groups with polynomial growth, Heintze’s [18] work on solvable Lie groups and homogeneous manifolds of negative curvature, Milnor’s [27] study of the curvature properties of left-invariant Riemannian metrics on Lie groups, the study of 3-dimensional model geometries and Dehn functions in the work of Epstein et al. [8] on automatic group, Pansu’s [30, 31] work on $L^p$ cohomology, de Cornulier’s [6] computation of the covering dimension of asymptotic cones of connected Lie groups, the study of quasi-isometries of certain solvable Lie groups by Eskin et al. [9], and Xie’s [36] quasi-isometric classification of negatively curved solvable Lie groups of the form $\mathbb {R}^n \rtimes \mathbb {R}$. Depending on the case to be treated, different tools are used in the classification problem, such as volume growth, Dehn functions, curvature and asymptotic cones of Riemannian manifolds.

#### Theorem 1.2

(Various authors) All connected real Lie groups of dimension at most three are classified up to quasi-isometries according to the following table:

**Table:** 

Class	Representatives
(1)	$\mathbb {T}^1$, $\mathbb {T}^2$, $\mathbb {T}^3$, $\mathrm {SU}(2)$, $\mathrm {SO}(3)$
(2)	$\mathbb {R}$, $\mathbb {R}\times \mathbb {T}^1$, $\mathbb {R}\times \mathbb {T}^2$
(3)	$\mathbb {R}^2$, $\mathbb {R}^2 \times \mathbb {T}^1$, $\mathrm {N}_3^{*}(\mathbb {R})$, $\{\mathrm {SE}(2)_k:k\in \mathbb {N}\}$
(4)	$\mathbb {R}^3$, $\widetilde{\mathrm {SE}}(2)$
(5)	$$\mathrm {N}_3(\mathbb {R})$$
(6)	$\widetilde{\mathrm {SL}}(2)$, ${\text {Aff}}^{+}(\mathbb {R})\times \mathbb {R}$
$(7_{\lambda })$ for $\lambda \in [-1,0)$	$\mathrm {D}_{\lambda }$
(8)	${\text {Aff}}^{+}(\mathbb {R})$, ${\text {Aff}}^{+}(\mathbb {R})\times \mathbb {T}^1$, $\{\mathrm {PSL}(2)_k:k\in \mathbb {N}\}$
(9)	$$\mathrm {J}$$
(10)	$\mathrm {D}_1$, $\{\mathrm {C}_{\lambda }:\lambda >0\}$
$(11_{\lambda })$ for $\lambda \in (0,1)$	$\mathrm {D}_{\lambda }$

We stress that the classes $(7_{\lambda })$ are distinct for different values of $\lambda$, and the same holds for $(11_{\lambda })$. In Sect. 3 we will explain how the above mentioned results by various authors can be combined to prove Theorem 1.2.

According to Theorem 1.2, two simply connected 3-dimensional Lie groups *G* and *H* (that are not isomorphic) are quasi-isometric to each other if and only if one of the following holds:

1. $$G,H\in \{\mathbb {R}^3,\widetilde{\mathrm {SE}}(2)\}$$
2. $$G,H \in \{\widetilde{\mathrm {SL}}(2),{\text {Aff}}^{+}(\mathbb {R})\times \mathbb {R}\}$$
3. $$G,H \in \{D_1\}\cup \{C_{\lambda }:\; \lambda >0\}$$.

In Proposition 2.2 we shall show that in all these cases, the two groups *G* and *H* may in fact be made isometric. By Proposition 2.1, this means that there exists a Riemannian manifold *M* on which both *G* and *H* act simply transitively by isometries. In fact, *M* may be taken equal to a Riemannian manifold that corresponds to one of the eight 3-dimensional model geometries by Thurston [35]:

- the Euclidean geometry in (1),
- the geometry of $\widetilde{\mathrm {SL}}(2)$ in (2),
- the hyperbolic geometry in (3),

see the discussion in Sect. 2.1, and in particular Remark 2.8 for (2). Thus we obtain the following result.

#### Theorem 1.3

If two non-isomorphic simply connected 3-dimensional Lie groups are quasi-isometric, then they may be made isometric to one of the eight Thurston geometries.

In Proposition 2.11 we shall show that without the assumption “simply connected”, it is not true in general that two connected, quasi-isometric Lie groups may be made isometric. Moreover, since the groups $\mathrm {PSL}(2)_k$, for different values of $k\in \mathbb {N}$, may all be made isometric to ${\text {Aff}}^{+}(\mathbb {R})\times \mathbb {T}^1$, but cannot be made isometric to each other, we have the following consequence.

#### Proposition 1.4

The relation “may be made isometric” is not transitive.

## Relations between groups

### Groups that may be made isometric

We begin the section with a basic observation about Lie groups that may be made isometric and carry on with a list of 3-dimensional Lie groups that may be made isometric.

#### Proposition 2.1

Two Lie groups *G* and *H* may be made isometric if and only if there exists a Riemannian manifold *M* on which both *G* and *H* act simply transitively by isometries.

#### Proof

Assume first that *G* and *H* possess Riemannian distances $d_G$ and $d_H$, respectively, for which there exists an isometry $\Psi : (G,d_G) \rightarrow (H,d_H)$. Take $M=H$ equipped with the Riemannian metric *g* that induces $d_H$. Clearly, *H* acts on *M* simply transitively by isometries, and the same is true for *G* with the action given by

$$\begin{aligned} G\times M \rightarrow M,\quad (g,m) \mapsto \Psi \circ L_g \circ \Psi ^{-1}(m), \end{aligned}$$

where $L_g$ denotes left translation by $g\in G$.

Conversely, assume that *G* and *H* act simply transitively on a manifold *M* with Riemannian distance *d*. Fix $x_0 \in M$ and define

$$\begin{aligned} d_G(g,g'):= d(g.x_0,g'.x_0),\quad g,g'\in G \end{aligned}$$

and

$$\begin{aligned} d_H(h,h'):= d(h.x_0,h'.x_0),\quad h,h'\in H. \end{aligned}$$

Since by assumption the actions of *G* and *H* on *M* are free, the above definition yields distance functions on *G* and *H*. From the compatibility of group actions and the fact that *G* and *H* act by isometries, we easily deduce that $d_G$ and $d_H$ are left-invariant. For instance, for *G*, we find for

$$\begin{aligned} d_G(g_0 g, g_0 g')= d(g_0.(gx_0),g_0 . (g'x_0)) = d(g.x_0,g'.x_0) = d_G(g,g'). \end{aligned}$$

Since the given actions by *G* and *H* on *M* are also transitive, for every $g\in G$ we find $h(g)\in H$ such that $g.x_0 = h(g).x_0$. This defines a map $(G,d_G)\rightarrow (H,d_H)$, $g\mapsto h(g)$, which is easily seen to be an isometry. $\square$

#### Proposition 2.2

Each of the following pairs consists of groups that may be made isometric:

1. $$(\mathbb {R}^3,\widetilde{\mathrm {SE}}(2))$$
2. $$(\mathbb {R}^2 \times \mathbb {T}^1,\mathrm {SE}(2)_k)$$ for every $$k\in \mathbb {N}$$
3. $$(\mathrm {SE}(2)_k,\mathrm {SE}(2)_{k'})$$ for all $$k,k'\in \mathbb {N}$$
4. $$(\widetilde{\mathrm {SL}}(2),{\text {Aff}}^{+}(\mathbb {R})\times \mathbb {R})$$
5. $$({\text {Aff}}^{+}(\mathbb {R})\times \mathbb {T}^1,\mathrm {PSL}(2)_k)$$ for every $$k\in \mathbb {N}$$
6. $$(D_1,C_{\lambda })$$ for every $$\lambda >0$$
7. $$(C_{\lambda },C_{\lambda '})$$ for all $$\lambda ,\lambda '>0$$.

#### Proof

It is well known that $\mathbb {R}^3$ and $\widetilde{\mathrm {SE}}(2)$ may be made isometric, see for instance [27, Corollary 4.8], [26, Theorem 2.14, (1-b)], and [23, §4]; or read the discussion later in this section. The statement that $\mathrm {SE}(2)_k$ may be made isometric to $\mathbb {R}^2 \times \mathbb {T}^1$ is Proposition 2.3. As a corollary, the groups $\mathrm {SE}(2)_k$ and $\mathrm {SE}(2)_{k'}$ for arbitrary $k,k'\in \mathbb {N}$ may be made isometric. Proposition 2.5 shows that $\widetilde{\mathrm {SL}}(2)$ and ${\text {Aff}}^{+}(\mathbb {R})\times \mathbb {R}$ may be made isometric. By Proposition 2.10, ${\text {Aff}}^{+}(\mathbb {R})\times \mathbb {T}^1$ may be made isometric to $\mathrm {PSL}(2)_k$ for every value of $k\in \mathbb {N}$.

The items (6) and (7) in Proposition 2.2 follow by curvature considerations. On the (simply connected) groups $D_1$ and on $C_{\lambda }$, $\lambda >0$, one can find a left-invariant Riemannian distance with constant negative sectional curvature: for $D_1$, this follows from Special Example 1.7 in Milnor’s article [27], for $C_{\lambda }$, $\lambda >0$, it is a consequence of [27, Theorem 4.11]; see also [26, Lemma 2.13 and Theorem 2.14, (1-a)] and [36, Introduction]. It is well known that every simply connected and complete Riemannian manifold with negative constant sectional curvature *K* is isometric to hyperbolic space in the respective dimension with sectional curvature *K*, hence all the groups $D_1$ and $C_{\lambda }$, $\lambda >0$ may be made isometric to hyperbolic 3-space, and thus also to each other. $\square$

We now provide the details for the results that have been used in the proof of Proposition 2.2 and for which no other reference has been given. The groups to be considered are $\widetilde{\mathrm {SE}}(2)$, $\widetilde{\mathrm {SL}}(2)$, and quotients thereof. The simply connected Lie group $\widetilde{\mathrm {SE}}(2)$ is isomorphic to $(\mathbb {R}^3,*)$, where

$$\begin{aligned} (x,y,\theta )*(x',y',\theta ')&= \left( \begin{pmatrix}x \\ y\end{pmatrix}+ \begin{pmatrix}\cos \theta &{}\quad -\,\sin \theta \\ \sin \theta &{}\quad \cos \theta \end{pmatrix}\begin{pmatrix}x' \\ y'\end{pmatrix},\theta + \theta '\right) \\&= (x+ x'\cos \theta -y'\sin \theta , y + x' \sin \theta + y'\cos \theta , \theta + \theta '). \end{aligned}$$

A direct computation shows that the Euclidean distance $d_E$ on $\mathbb {R}^3$ is left-invariant with respect to $*$, and hence $\mathbb {R}^3$ and $\widetilde{\mathrm {SE}}(2)$ may be made isometric. It is easy to verify that the sets $(N_k,*)$, $k\in \mathbb {N}$, given by

$$\begin{aligned} N_k = \{(0,0,2\pi k m): m\in \mathbb {Z}\}, \end{aligned}$$

are exactly the discrete normal subgroups of $\widetilde{\mathrm {SE}}(2)$. Every $k\in \mathbb {N}$ gives thus rise to a multiply connected Lie group

$$\begin{aligned} \mathrm {SE}(2)_k:= \widetilde{\mathrm {SE}}(2)/ N_k. \end{aligned}$$

The center of $\mathrm {SE}(2)_k$ contains exactly *k* elements, which shows that $\mathrm {SE}(2)_k$ is not isomorphic to $\mathrm {SE}(2)_l$ for $k\ne l$. Moreover, $\mathrm {SE}(2)_k$ is isomorphic to $(\mathbb {R}^2 \times (\mathbb {R}/2\pi k \mathbb {Z}),*_k)$, where

$$\begin{aligned} (x,y,\theta ) *_k (x',y',\theta ') = (x+ x'\cos \theta -y'\sin \theta , y + x' \sin \theta + y'\cos \theta , \theta + \theta '). \end{aligned}$$

#### Proposition 2.3

For every $k\in \mathbb {N}$, the group $\mathrm {SE}(2)_k$ may be made isometric to the standard round cylinder $\mathbb {R}^2 \times \mathbb {R}/\mathbb {Z}$.

#### Proof

We construct a left-invariant distance on $\mathrm {SE}(2)_k$, by setting

2.1 $$\begin{aligned} d_{SE(2)_k}((x,y,\theta ),(x',y',\theta ')):= \sqrt{\Vert (x,y)-(x',y')\Vert ^2+( {(2\pi k)}^{-1}d_{\mathbb {R}/ 2\pi k \mathbb {Z}}(\theta ,\theta '))^2} \end{aligned}$$

for $(x,y,\theta )$ and $(x',y',\theta ')$ in $\mathbb {R}^2 \times (\mathbb {R}/ 2\pi k \mathbb {Z})$. Here

$$\begin{aligned} d_{\mathbb {R}/ 2\pi k \mathbb {Z}}(\theta ,\theta '):= \min _{m\in \mathbb {Z}} \{|2\pi k m - (\theta -\theta ')|\}, \end{aligned}$$

Then the map $\Psi : \mathbb {R}^2 \times \mathbb {R}/\mathbb {Z}\rightarrow \mathbb {R}^2 \times (\mathbb {R}/ 2\pi k \mathbb {Z})$ given by

$$\begin{aligned} \Psi (x,y,{\theta }) = (x,y,2 \pi k\theta ) \end{aligned}$$

provides an isometry between $\mathbb {R}^2 \times \mathbb {R}/\mathbb {Z}$ and $\mathrm {SE}(2)_k$. $\square$

We now turn our attention to $\widetilde{\mathrm {SL}}(2)$ and its quotients. Since $\widetilde{\mathrm {SL}}(2)$ is a simple Lie group, [5, Corollary 3.11] is useful.

#### Theorem 2.4

(Cowling et al.) Let *G* be a connected semisimple Lie group and let $G=ANK$ be its Iwasawa decomposition. Write *K* as $V \times K'$, where *V* is a vector group and $K'$ is compact. Then *G* may be made isometric to the direct product $AN \times V \times K'$.

If *K* is compact, then *G* may be made isometric to $AN \times K$. A condition which ensures the compactness of *K* for a given semisimple Lie group is that *G* has finite center, see [12, p.160 in Chapter 4]. A connected semisimple Lie group that is linear has finite center, see for instance [12, Chapter 1, §5].

The Iwasawa decomposition of $\widetilde{\mathrm {SL}}(2)$ is *ANK*, where *A* and *N* are the following matrix groups

$$\begin{aligned} A= \left\{ \begin{pmatrix}e^t &{}\quad 0 \\ 0&{}\quad e^{-t}\end{pmatrix}:\; t\in \mathbb {R}\right\} ,\quad N= \left\{ \begin{pmatrix}1 &{}\quad x \\ 0&{}\quad 1\end{pmatrix}:\; x\in \mathbb {R}\right\} , \end{aligned}$$

and *K* is isomorphic to $\mathbb {R}$. More precisely, the Iwasawa decomposition is given by the diffeomorphism

$$\begin{aligned} \phi : \mathbb {R}^3 \rightarrow \widetilde{\mathrm {SL}}(2) \end{aligned}$$

so that $\phi (0,0,0)=\mathrm {I}$ and

$$\begin{aligned} (\pi \circ \phi )(t,x,\theta ) = \begin{pmatrix}e^t &{}\quad 0 \\ 0&{}\quad e^{-t}\end{pmatrix} \begin{pmatrix}1 &{}\quad x \\ 0&{}\quad 1\end{pmatrix} \begin{pmatrix}\cos \theta &{}\quad \sin \theta \\ -\,\sin \theta &{}\quad \cos \theta \end{pmatrix}, \end{aligned}$$

where $\pi :\widetilde{\mathrm {SL}}(2) \rightarrow \mathrm {SL}(2)$ is the universal covering projection. Note that *AN* is isomorphic to the orientation-preserving affine maps of the real line, that is, to ${\text {Aff}}^{+}(\mathbb {R})$.

Theorem 2.4 applied to the Iwasawa decomposition of $\widetilde{\mathrm {SL}}(2)$ yields the following statement.

#### Proposition 2.5

The groups $\widetilde{\mathrm {SL}}(2)$ and ${\text {Aff}}^{+}(\mathbb {R})\times \mathbb {R}$ may be made isometric.

#### Remark 2.6

The group ${\text {Aff}}^{+}(\mathbb {R})$ admits a left-invariant metric of constant negative sectional curvature (see for instance [27, Special Example 1.7]) and hence, by the same reasoning as in the proof of Proposition 2.2, it may be made isometric to the hyperbolic plane $\mathbb {H}^2$. The quasi-isometric, or even bi-Lipschitz, equivalence of $\mathbb {H}^2\times \mathbb {R}$ and $\widetilde{\mathrm {SL}}(2)$ was proved earlier by Rieffel [33] in her Ph.D. thesis. The idea of the construction is explained in [22, §2]. To set the stage, we follow [34, p. 462] and observe that the standard Riemannian metric on $\mathbb {H}^2$ induces a natural Riemannian metric on $T\mathbb {H}^2$ in such a way that for every isometry $f:\mathbb {H}^2 \rightarrow \mathbb {H}^2$, the differential $df: T\mathbb {H}^2 \rightarrow T\mathbb {H}^2$ is an isometry as well. Since the unit tangent bundle $UT\mathbb {H}^2$ is a submanifold of $T\mathbb {H}^2$, it inherits a Riemannian metric from $T\mathbb {H}^2$, and as $UT(\mathbb {H}^2)$ may be identified with $\mathrm {PSL}(2)$, this metric lifts to $\widetilde{\mathrm {SL}(2)}$. One can show that the thus obtained Riemannian metric on $\widetilde{\mathrm {SL}(2)}$ is left-invariant, see [34, p. 464].

To prove the bi-Lipschitz equivalence of $\mathbb {H}^2\times \mathbb {R}$ and $\widetilde{\mathrm {SL}}(2)$, one constructs a map

$$\begin{aligned} f:UT( {\mathbb {H}^2})\rightarrow {\mathbb {H}^2} \times {\mathbb {S}}^1,\quad f(v):=(x,\phi (v)), \end{aligned}$$

as follows: first, one fixes a point $p_0\in {\mathbb {H}^2}$, then, for $v\in UT_x( {\mathbb {H}^2})$, the vector $\phi (v)\in UT_{p_0}( {\mathbb {H}^2})$ is obtained by parallel transporting *v* along the geodesic segment $[xp_0]$. One then proves that *f* is bi-Lipschitz; see [21, Proposition 3.10], and [7, IV.48] for more details. Since *f* lifts to a bi-Lipschitz map between universal covers, see Proposition 2.13, this reasoning shows that $\mathbb {H}^2\times \mathbb {R}$ and $\widetilde{\mathrm {SL}}(2)$ are bi-Lipschitz equivalent, and in particular quasi-isometrically equivalent.

#### Remark 2.7

By Proposition 2.5, the group $\widetilde{\mathrm {SL}}(2)$ may be made isometric to ${\text {Aff}}^{+}(\mathbb {R})\times \mathbb {R}$. Moreover, according to Remark 2.6, the group ${\text {Aff}}^{+}(\mathbb {R})\times \mathbb {R}$ may be made isometric to $\mathbb {H}^2 \times \mathbb {R}$ with the standard metric. However, this does not imply that $\widetilde{\mathrm {SL}}(2)$ can be made isometric to the standard $\mathbb {H}^2 \times \mathbb {R}$, and indeed this is not the case: An isometry between the $\mathbb {H}^2 \times \mathbb {R}$ and $\widetilde{\mathrm {SL}}(2)$ with a left-invariant distance would induce a free transitive isometric action of $\widetilde{\mathrm {SL}}(2)$ on $\mathbb {H}^2 \times \mathbb {R}$. Notice that every isometry *f* of $\mathbb {H}^2 \times \mathbb {R}$ sends a set of the form $\mathbb {H}^2 \times \{p\}$ to the set $\mathbb {H}^2 \times \{f(p)\}$, since these sets are the leaves of the foliation integrating the planes of sectional curvature $-1$. Thus, if $\widetilde{\mathrm {SL}}(2)$ acts by isometry on $\mathbb {H}^2 \times \mathbb {R}$, then the induced action on $\mathbb {R}$ would be by translations, since $\widetilde{\mathrm {SL}}(2)$ is connected. At the same time, the action would have to be trivial since the Lie algebra of $\widetilde{\mathrm {SL}}(2)$ is simple, so it could not act transitively on $\mathbb {H}^2 \times \mathbb {R}$. See also [34, Section 5].

Since the groups $\widetilde{\mathrm {SL}}(2)$ and ${\text {Aff}}^{+}(\mathbb {R})\times \mathbb {R}$ may be made isometric, one might wonder if there is a “standard” Riemannian manifold to which they may both be made isometric. According to Remark 2.7, this manifold cannot be the standard $\mathbb {H}^2 \times \mathbb {R}$, but it turns out that $\widetilde{\mathrm {SL}}(2)$ endowed with the metric corresponding to one of the Thurston geometries has the desired property; see Remark 2.8 below.

Consider the left-invariant Riemannian metric $g_{\widetilde{\mathrm {SL}}(2)}$ on $X:=\widetilde{\mathrm {SL}}(2)$ that arises from the identification of $\mathrm {PSL}(2)$ with the unit tangent bundle $UT(\mathbb {H}^2)$ as described in Remark 2.6 and let $G:=\mathrm {Isome}(\widetilde{\mathrm {SL}}(2))$ be the corresponding isometry group. Then (*X*, *G*) is one of the eight three-dimensional model geometries of Thurston [35, Theorem 3.8.4]. Clearly, $\widetilde{\mathrm {SL}}(2)$ acts transitively by isometries on $(X,g_{\widetilde{\mathrm {SL}}(2)})$. The following remark shows that the same is true for ${\text {Aff}}^{+}(\mathbb {R})\times \mathbb {R}$. According to Proposition 2.1, this also provides another proof for Proposition 2.5.

#### Remark 2.8

The group ${\text {Aff}}^{+}(\mathbb {R})\times \mathbb {R}$ acts simply transitively by isometries on *X* endowed with the Riemannian metric that corresponds to Thurston’s model geometry on $\widetilde{\mathrm {SL}}(2)$. To see this, consider the group $G:=\mathrm {Isome}(\widetilde{\mathrm {SL}}(2))$, which has been discussed in [34, p. 464 ff]. It has been shown that *G* consists of two components, say $\Gamma$ and $\Gamma '$. The identity component $\Gamma$ is a 4-dimensional Lie group generated by the actions of $\mathbb {R}$ and $\widetilde{\mathrm {SL}}(2)$ on *X*. The action of $\widetilde{\mathrm {SL}(2)}$ is immediate, and according to the Iwasawa decomposition, it yields in particular an action of ${\text {Aff}}^{+}(\mathbb {R})$ on *X*. To explain the action of $\mathbb {R}$, it is useful to see *X* as a line bundle over $\mathbb {H}^2$. The center of $\widetilde{\mathrm {SL}}(2)$, which is isomorphic to the additive group $\mathbb {Z}$, acts on *X* by preserving the line bundle structure and covering the identity map of $\mathbb {H}^2$. This action extends to an action of $\mathbb {R}$ on *X* by translation of the vertical fibers [this action arises as an action of $S^1$ on $UT(\mathbb {H}^2)$ which covers the identity of $\mathbb {H}^2$ and rotates each fibre by the same angle]. Since the action of $\mathbb {R}$ commutes with the action of $\widetilde{\mathrm {SL}}(2)$ [and thus of ${\text {Aff}}^{+}(\mathbb {R})$], we obtain that ${\text {Aff}}^{+}(\mathbb {R})\times \mathbb {R}$ acts by isometries on *X*. Moreover, since ${\text {Aff}}^{+}(\mathbb {R})\times \mathbb {R}$ acts transitively on the base manifold $\mathbb {H}^2$ of *X*, and $\mathbb {R}$ acts by translation on the vertical fibers, we see that ${\text {Aff}}^{+}(\mathbb {R})\times \mathbb {R}$ acts transitively on *X*. Finally, we argue that the action is free. Assume that $(g,s).x= x$ for some $g\in {\text {Aff}}^{+}(\mathbb {R})$, $s\in \mathbb {R}$ and $x\in X$. Then, since the action of $\mathbb {R}$ covers the identity map of $\mathbb {H}^2$, it follows that *g*.*x* and *x* must lie in the same vertical fibre of *X*. As the action of ${\text {Aff}}^{+}(\mathbb {R})$ on *X* is induced by a free action of ${\text {Aff}}^{+}(\mathbb {R})$ on $\mathbb {H}^2$, it follows that $g=e$, as desired. Moreover, $s=0$ since the action of $\mathbb {R}$ is free. This shows that ${\text {Aff}}^{+}(\mathbb {R})\times \mathbb {R}$ acts simply transitively by isometries on $(X,g_{\widetilde{\mathrm {SL}}(2)})$.

#### Remark 2.9

As the classification in Theorem 1.2 shows, already in dimension 3 the property of admitting a lattice (i.e., a discrete subgroup of cofinite volume) is not a quasi-isometric invariant. For example, the group ${\text {Aff}}^{+}(\mathbb {R})\times \mathbb {T}^1$ is not unimodular by [27, Lemma 6.3] and hence cannot have lattices (see [27, Section 6] or [1, Proposition 2.4.2]), yet it is quasi-isometrically equivalent to $\mathrm {SL}(2)=\mathrm {SL}(2, \mathbb {R})$, which admits the lattice $\mathrm {SL}(2,\mathbb {Z})$.

For $k \in \mathbb {N}$, the Iwasawa decomposition of $\mathrm {PSL}(2)_k$ is

$$\begin{aligned} \mathrm {PSL}(2)_k = ANK_k, \end{aligned}$$

where $K_k$ is the *k*-fold cover of the projective special orthogonal group $\mathrm {PSO}(2)$.

Theorem 2.4 applied to the Iwasawa decomposition of $\mathrm {PSL}(2)_k$ yields the following result.

#### Proposition 2.10

For every $k\in \mathbb {N}$, the group $\mathrm {PSL}(2)_k$ may be made isometric to ${\text {Aff}}^{+}(\mathbb {R})\times \mathbb {T}^1$.

### Bi-Lipschitz groups

#### Proposition 2.11

The groups $\mathrm {PSL}(2)_k$ and $\mathrm {PSL}(2)_{k'}$ for different values of $k,k'\in \mathbb {N}$ are bi-Lipschitz equivalent, but cannot be made isometric.

The bi-Lipschitz equivalence of $\mathrm {PSL}(2)_k$ and $\mathrm {PSL}(2)_{k'}$ follows easily from Proposition 2.10, but to show that these groups cannot be made isometric, we use [13, Theorem 2.2] by Gordon, which we restate here for the reader’s convenience.

Assume that *A* is a connected Lie group with a connected subgroup *G*. Choose Levi factors $G_s$ and $A_s$ of *G* and *A*, respectively, such that $G_s \subset A_s$, and denote by $\mathfrak {g}_s$ and $\mathfrak {a}_s$ the Lie algebras of $G_s$ and $A_s$. By definition, the Lie algebras $\mathfrak {g}_s$ and $\mathfrak {a}_s$ are semisimple and thus a direct sum of simple Lie algebras, some of which may be compact and others not. This leads to the direct sum decomposition

$$\begin{aligned} \mathfrak {g}_s= \mathfrak {g}_{nc} \oplus \mathfrak {g}_c, \end{aligned}$$

where $\mathfrak {g}_c$ is the direct sum of all compact simple ideals of $\mathfrak {g}_s$ and $\mathfrak {g}_{nc}$ is the direct sum of the remaining simple ideals. In the same way, one decomposes $\mathfrak {a}_s= \mathfrak {a}_{nc} \oplus \mathfrak {a}_c$. By $G_{nc}$ and $A_{nc}$ we denote the connected subgroups of *A* with Lie algebras $\mathfrak {g}_{nc}$ and $\mathfrak {a}_{nc}$, respectively.

#### Theorem 2.12

(Gordon) Assume that *A* is a connected Lie group with a connected subgroup *G* whose radical is nilpotent. Suppose further that there exists a compact subgroup *K* of *A* such that $A= GK$. Then $A_{nc}=G_{nc}$.

With this theorem at hand, we can prove Proposition 2.11.

#### Proof of Proposition 2.11

By Proposition 2.10, both $\mathrm {PSL}(2)_k$ and $\mathrm {PSL}(2)_{k'}$ may be made isometric to ${\text {Aff}}^{+}(\mathbb {R})\times \mathbb {T}^1$. Thus there exist left-invariant Riemannian distances, say $d_k$ and $d_{k'}$ on ${\text {Aff}}^{+}(\mathbb {R})\times \mathbb {T}^1$, as well as *d* on $\mathrm {PSL}(2)_k$ and $d'$ on $\mathrm {PSL}(2)_{k'}$ such that $(\mathrm {PSL}(2)_k,d)$ is isometric to $( {\text {Aff}}^{+}(\mathbb {R})\times \mathbb {T}^1,d_k)$ and $(\mathrm {PSL}(2)_{k'},d')$ is isometric to $({\text {Aff}}^{+}(\mathbb {R})\times \mathbb {T}^1,d_{k'})$. Since $d_k$ and $d_{k'}$ are bi-Lipschitz equivalent, it follows that $\mathrm {PSL}(2)_k$ and $\mathrm {PSL}(2)_{k'}$ are bi-Lipschitz equivalent.

Next we show that $\mathrm {PSL}(2)_k$ and $\mathrm {PSL}(2)_{k'}$ cannot be made isometric. For $k\in \mathbb {N}$, we fix a left-invariant Riemannian distance $d_G$ on $G:= \mathrm {PSL}(2)_k$ and we let *A* be the isometry group of $(G,d_G)$. Then $A= GK$ as in Theorem 2.12, with $K = \mathrm {Stab}(e)\cap A$, where $\mathrm {Stab}(e)$ denotes the stabilizer of the identity in *G*. Since *G* is simple, its radical is trivial and hence nilpotent and moreover, $G_{nc}=G$. It follows by Theorem 2.12 that $G = G_{nc} = A_{nc}$. The same reasoning applies for $k'$ instead of *k*, so that we obtain $G'=A'_{nc}$ for $G'=\mathrm {PSL}(2)_{k'}$ and $A'$ the isometry group of $(G',d_{G'})$. Now if $(G,d_G)$ and $(G',d_{G'})$ were isometric, then *A* would be isomorphic to $A'$ with an isomorphism given by conjugation via the isometry between $(G,d_G)$ and $(G',d_{G'})$. This would imply that $\mathrm {PSL}(2)_k = A_{nc}$ is isomorphic to $A'_{nc}= \mathrm {PSL}(2)_{k'}$, which is possible only if $k=k'$ [otherwise the centers of $\mathrm {PSL}(2)_k$ and $\mathrm {PSL}(2)_{k'}$ have different cardinality and hence the groups cannot be isomorphic]. $\square$

### Quasi-isometrically homeomorphic groups

We now consider multiply connected groups that are homeomorphic via a quasi-isometry but not bi-Lipschitz equivalent. The latter fact will be proved by contradiction: if there existed a bi-Lipschitz homeomorphism between the groups it would lift to a bi-Lipschitz homeomorphism of the universal covers according to Proposition 2.13. We first recall some basics from covering theory.

Assume that *G* is a simply connected Lie group equipped with a left-invariant Riemannian metric *g*. If *N* is a discrete normal subgroup of *G*, then *G*/*N* is a connected Lie group which admits a unique left-invariant Riemannian metric $g_{G/N}$ so that $\pi : (G,g) \rightarrow (G/N,g_{G/N})$ becomes a Riemannian covering, that is, a covering map which is locally isometric.

#### Proposition 2.13

For $i\in \{1,2\}$, let $G_i$ be a simply connected Lie group endowed with a left-invariant Riemannian distance and let $\pi _i: (G_i,g_i) \rightarrow (G_i/N_i,g_{G_i/N_i})$ be a Riemannian covering as above. Then every bi-Lipschitz homeomorphism $f: G_1/N_1 \rightarrow G_2/N_2$ lifts to a bi-Lipschitz homeomorphism $\widetilde{f}: G_1 \rightarrow G_2$, where ‘bi-Lipschitz’ refers to the Riemannian distances induced by the respective Riemannian metrics.

#### Proof

Let $f: G_1/N_1 \rightarrow G_2/N_2$ be bi-Lipschitz. Since *f* is a homeomorphism and $G_1$ is simply connected, the composition $f\circ \pi _1: G_1 \rightarrow G_2/N_2$ is a universal cover of $G_2/N_2$, as is the map $\pi _2: G_2 \rightarrow G_2/N_2$. It follows from the uniqueness theorem for universal covers, see for instance [10, Corollary 13.6] or [24, I, §11], that there exists a homeomorphism $\widetilde{f}: G_1 \rightarrow G_2$ with $\pi _2 \circ \widetilde{f} = f \circ \pi _1$. Since *f* is bi-Lipschitz and $\pi _1$, $\pi _2$ are local isometries, the map $\widetilde{f}$ is uniformly locally bi-Lipschitz, as is its inverse. Finally, since $G_1$ and $G_2$ are geodesic, $\widetilde{f}$ is bi-Lipschitz as claimed. $\square$

#### Proposition 2.14

Each of the following pairs consists of quasi-isometrically homeomorphic groups that are not bi-Lipschitz equivalent:

1. $$(\mathbb {R}^2 \times \mathbb {T}^1,\mathrm {N}_3^{*}(\mathbb {R}))$$
2. $$(\mathrm {SE}(2)_k,\mathrm {N}_3^{*}(\mathbb {R}))$$, for every $$k\in \mathbb {N}$$.

#### Proof

Once we know that $\mathbb {R}^2 \times \mathbb {T}^1$ and $\mathrm {N}_3^{*}(\mathbb {R})$ are equivalent via a quasi-isometric but not a bi-Lipschitz homeomorphism, the same statements follow for $SE(2)_k$ and $\mathrm {N}_3^{*}(\mathbb {R})$ by Proposition 2.2, (2). Thus it suffices to prove Part (1) of Proposition 2.14.

In order to show that the groups $\mathrm {N}_3^{*}(\mathbb {R})$ and $\mathbb {R}^2 \times \mathbb {T}^1$ are quasi-isometric via a homeomorphism, it is convenient to choose, as we may, coordinates (*x*, *y*, *z*) on $\mathrm {N}_3(\mathbb {R})$ so that for all (*x*, *y*, *z*) and $(x',y',z')$, we have

$$\begin{aligned} (x,y,z) \cdot (x',y',z')= (x + x', y + y', z + z' + 2 y {x'} - 2 x {y'}). \end{aligned}$$

Without loss of generality we may assume that $\mathrm {N}_3^{*}(\mathbb {R})$ is the quotient of $\mathrm {N}_3(\mathbb {R})$ by the cyclic group generated by the element $Z=(0,0,1)$. The Lie group $\mathrm {N}_3(\mathbb {R}) /\langle Z \rangle$ is diffeomorphic to $\mathbb {R}^2 \times \mathbb {T}^1$. We see that $\mathbb {Z}^2$ can be identified with a subgroup of the groups $\mathrm {N}_3(\mathbb {R}) /\langle Z \rangle$ and $\mathbb {R}^2 \times \mathbb {T}^1$, respectively, which in both cases acts co-compactly. Moreover, for these particular models, the identity map of $\mathbb {R}^2 \times \mathbb {T}^1$ provides a quasi-isometric homeomorphism between $\mathrm {N}_3^{*}(\mathbb {R})$ and $\mathbb {R}^2 \times \mathbb {T}^1$.

Assume towards a contradiction that there exists a biLipschitz map $f: \mathbb {R}^2 \times \mathbb {T}^1 \rightarrow \mathrm {N}_3^{*}(\mathbb {R})$. It follows from Proposition 2.13 that there would exists a bi-Lipschitz homeomorphism $\widetilde{f}: \mathbb {R}^3 \rightarrow \mathrm {N}_3(\mathbb {R})$. This is known to be false, for instance because $\mathbb {R}^3$ has volume growth of order 3, whereas the volume of balls in $\mathrm {N}_3(\mathbb {R})$ grows with order 4 at large. We have thus proven that $\mathrm {N}_3^{*}(\mathbb {R})$ is not bi-Lipschitz equivalent to $\mathbb {R}^2 \times \mathbb {T}^1$. $\square$

### Quasi-isometric groups

#### Proposition 2.15

Each of the following pairs consists of quasi-isometrically equivalent groups that are not equivalent via a quasi-isometric homeomorphism:

1. (*G*, *H*) for distinct $$G,H \in \{\mathbb {T}^1, \mathbb {T}^2, \mathbb {T}^3, \mathrm {SU}(2), \mathrm {SO}(3)\}$$
2. (*G*, *H*) for distinct $$G,H \in \{\mathbb {R}, \mathbb {R}\times \mathbb {T}^1, \mathbb {R}\times \mathbb {T}^2\}$$
3. $$(\mathbb {R}^2,\mathbb {R}^2 \times \mathbb {T}^1)$$
4. $$({\text {Aff}}^{+}(\mathbb {R}), {\text {Aff}}^{+}(\mathbb {R})\times \mathbb {T}^1)$$
5. $$(\mathbb {R}^2,\mathrm {N}_3^{*}(\mathbb {R}))$$
6. $$(\mathbb {R}^2,\mathrm {SE}(2)_k)$$, for every $$k\in \mathbb {N}$$
7. $$({\text {Aff}}^{+}(\mathbb {R}),\mathrm {PSL}(2)_k)$$, for every $$k\in \mathbb {N}$$.

#### Proof

The groups appearing on the same line in Proposition 2.15 are topologically distinct and hence cannot be equivalent via a quasi-isometric homeomorphism. Indeed, denoting by “$\simeq$” equivalence via a diffeomorphism of manifolds, we have:

1. $$\mathbb {T}^1\simeq {\mathbb {S}}^1$$, $$\mathbb {T}^2 \simeq {\mathbb {S}}^1 \times {\mathbb {S}}^1$$, $$\mathbb {T}^3 \simeq {\mathbb {S}}^1 \times {\mathbb {S}}^1 \times {\mathbb {S}}^1$$, $$\mathrm {SU}(2)\simeq {\mathbb {S}}^3$$, $$\mathrm {SO}(3)\simeq P\mathbb {R}^3$$
2. $$\mathbb {R}$$, $$\mathbb {R}\times \mathbb {T}^1\simeq \mathbb {R}\times {\mathbb {S}}^1$$, $$\mathbb {R}\times \mathbb {T}^2\simeq \mathbb {R}\times {\mathbb {S}}^1\times {\mathbb {S}}^1$$
3. $$\mathbb {R}^2$$ and $$\mathbb {R}^2 \times \mathbb {T}^1\simeq \mathbb {R}^2 \times {\mathbb {S}}^1$$
4. $${\text {Aff}}^{+}(\mathbb {R})\simeq \mathbb {R}^2$$ and $${\text {Aff}}^{+}(\mathbb {R})\times \mathbb {T}^1\simeq \mathbb {R}^2 \times {\mathbb {S}}^1$$
5. $$\mathbb {R}^2$$ and $$\mathrm {N}_3^{*}(\mathbb {R})\simeq \mathbb {R}^2 \times {\mathbb {S}}^1$$
6. $$\mathbb {R}^2$$ and $$\mathrm {SE}(2)_k\simeq \mathbb {R}^2 \times {\mathbb {S}}^1$$
7. $${\text {Aff}}^{+}(\mathbb {R})\simeq \mathbb {R}^2$$ and $$\mathrm {PSL}(2)_k\simeq \mathbb {R}^2 \times {\mathbb {S}}^1$$.

It remains to show that groups appearing on the same line are quasi-isometrically equivalent, even if they are not homeomorphic. First, the groups $\mathbb {T}^1$, $\mathbb {T}^2$, $\mathbb {T}^3$, $\mathrm {SU}(2)$, and $\mathrm {SO}(3)$ are trivially quasi-isometrically equivalent because they are compact.

Second, the groups $\mathbb {R}$, $\mathbb {R}\times \mathbb {T}^1$, and $\mathbb {R}\times \mathbb {T}^2$ are clearly quasi-isometrically equivalent. More generally, $\mathbb {R}\times K$ is quasi-isometric to $\mathbb {R}\times K'$ for arbitrary compact Lie groups *K* and $K'$, as one can see by arguing componentwise. For the same reason, $\mathbb {R}^2$ and $\mathbb {R}^2 \times \mathbb {T}^1$ are quasi-isometrically equivalent, and so are ${\text {Aff}}^{+}(\mathbb {R})$ and ${\text {Aff}}^{+}(\mathbb {R})\times \mathbb {T}^1$. Having established the quasi-isometric equivalence in the cases (1)–(4), the remaining cases follow by transitivity. Indeed, the information from Propositions 2.14, 2.2, and 2.10 can be used to deduce that the groups in (5), (6), and (7) are quasi-isometrically equivalent, once this has been established for the groups in (3) and (4). $\square$

## Conclusion of the quasi-isometric classification

In Sect. 2 we have identified pairs of Riemannian Lie groups that are quasi-isometrically equivalent. In this section we show that all remaining pairs of at most three-dimensional connected Lie groups are quasi-isometrically distinct, thus establishing Theorem 1.2. The proof uses the following quasi-isometric invariants of connected Riemannian Lie groups:

- *degree of polynomial volume growth*
- *polynomial volume growth* (or equivalently by [17, 19]: type R)
- *Gromov hyperbolicity* [15], see also e.g. [29, Theorem 3.1.11]
- *covering dimension of asymptotic cones* [6].

Besides these general quasi-isometry invariants, we also rely on quasi-isometric classification results for connected Riemannian Lie groups of a specific form:

- for Gromov hyperbolic connected Riemannian Lie groups (which are proper metric spaces): *topology of the boundary* [15], see also e.g. [20, Proposition 2.20]
- for simply connected Riemannian manifolds of negative or zero curvature: $L^p$ *cohomology* [16]
- [30, Corollaire 1] and [36, Corollary 1.3] for $\mathbb {R}^n \rtimes _A \mathbb {R}$ with $A\in \mathrm {Mat}(n\times n)$ having only eigenvalues with positive real parts
  (in our notation this applies to: *J*, $D_{\lambda }$ for $0<\lambda \le 1$, $C_{\lambda }$ for $\lambda >0$)
- [9, Theorem 1.3] for $\mathrm {Sol}(m,n)$, the solvable Lie groups $\mathbb {R}^2 \rtimes \mathbb {R}$, where $\mathbb {R}$ acts by $z\cdot (x,y)= (e^{mz}x,e^{-nz}y)$, for $m>n>0$ using *coarse differentiation*
  (in our notation: $\mathrm {Sol}(1,-\lambda )=D_{\lambda }$ for $-1<\lambda <0$)

### Proof of Theorem 1.2

We first discuss why the listed classes are quasi-isometrically distinct.

*The groups in classes (1)–(5)* are the only groups of type R, as can be seen from an explicit description of the Bianchi classification of Lie algebras, as given for instance in [12, Chapter 7, §1.1]. The individual classes are divided according to the degree $d\in \{0,1,2,3,4\}$ of polynomial volume growth.

*The groups in classes (6) and* $(7_{\lambda })$ have exponential growth but are not Gromov hyperbolic: for the groups in class (6) this is easy to see since ${\text {Aff}}^{+}(\mathbb {R})\times \mathbb {R}$ can be endowed with a left-invariant Riemannian metric such that it contains an isometrically embedded copy of $\mathbb {R}^2$. The proof that $D_{\lambda }$ is not hyperbolic for $\lambda <0$ is given below in Proposition 3.1.

We now show that (6) and $(7_{\lambda })$ are distinct classes. The group ${\text {Aff}}^{+}(\mathbb {R})\times \mathbb {R}$ is not quasi-isometrically equivalent to any $D_{\lambda }$ since the covering dimension of the asymptotic cone of $D_{\lambda }$ is 1 for every $\lambda$, while ${\text {Aff}}^{+}(\mathbb {R})\times \mathbb {R}$ has cone dimension 2 by [6, Theorem 1.1].

To distinguish the classes $(7_{\lambda })$ for different values of $\lambda \in [-1,0)$, take $-1\le \lambda _1< \lambda _2<0$. If $\lambda _1\ne -1$, then $D_{\lambda _1}$ is not quasi-isometric to $D_{\lambda _2}$ by [9, Theorem 1.3]. If $\lambda _1=-1$, then $D_{\lambda _1}= D_{-1}$ is the Lie group of the Solv geometry, which by [25, Section 2] and [3, Section 3] admits a cocompact lattice of the form $\mathbb {Z} \ltimes \mathbb {Z}^2$, while there does not exist any finitely generated group quasi-isometric to $D_{\lambda _2}$ by [9, Theorem 1.2].

*The groups in classes (8)*–$(11_{\lambda })$ are Gromov hyperbolic: since ${\text {Aff}}^{+}(\mathbb {R})$, *J*, and $D_\lambda$ for $\lambda \in (0,1]$ are all of the form $\mathbb {R}^n \rtimes _A \mathbb {R}$ for a matrix *A* whose eigenvalues all have positive real parts, it follows from [18, Theorem 3] that each of these groups admits a left-invariant Riemannian metric with negative sectional curvature bounded away from zero. Finally, a simply connected complete Riemannian manifold with negative curvature bounded away from zero is Gromov hyperbolic, see for instance [11, p. 52, Corollaire 10]. While the groups in (8) have ${\mathbb {S}}^1$ as visual boundary, the groups in (9)-$(11_{\lambda })$ have ${\mathbb {S}}^2$.

All groups *J*, $D_{\lambda }$ ($\lambda \in (0,1]$) are of the form $\mathbb {R}^2 \rtimes _A \mathbb {R}$ with *A* equal to $$\left( \begin{array}{cc}1&{}1 \\ 0&{}1\end{array}\right) $$ or $$\left( \begin{array}{cc}1&{}0\\ {0}&{}\lambda \end{array}\right) $$, $\lambda \in (0,1]$. It is a special case of [30, Corollaire 1], proved by means of $L^p$ cohomology, that two groups in the family $D_{\lambda }$, $D_{\lambda '}$, $\lambda ,\lambda ' \in (0,1]$ are quasi-isometrically equivalent if and only if they are isomorphic, that is, if and only if $\lambda = \lambda '$. The quasi-isometric classification of all negatively curved $\mathbb {R}^n \rtimes \mathbb {R}$ has been completed in [36]. As a special case of [36, Corollary 1.3], if *A* and *B* are $2\times 2$ matrices whose eigenvalues all have positive real parts, then the two groups $\mathbb {R}^2 \rtimes _A \mathbb {R}$ and $\mathbb {R}^2 \rtimes _B \mathbb {R}$ are quasi-isometric if and only if there exists $s>0$ such that *A* and *sB* have same real part Jordan form. This shows in particular that *J* cannot be quasi-isometric to any $D_{\lambda }$, $\lambda \in (0,1]$, and $D_{\lambda }$ is quasi-isometric to $D_{\lambda '}$ only if $\lambda = \lambda '$. The previous discussion also covers the groups $\{C_{\lambda }:\; \lambda >0\}$, which are quasi-isometric to $D_1$.

Except for $(7_{\lambda })$ and $(11_{\lambda })$, which represent uncountably many different classes, all the groups listed on one line in the table in Theorem 1.2 are quasi-isometrically equivalent: this follows from Propositions 2.2, 2.11, 2.14, and 2.15. $\square$

We now discuss the proof of one result which has been used in the quasi-isometric classification.

### Proposition 3.1

The Lie groups $D_{\lambda }$, $\lambda \in [-1,0)$, are not Gromov hyperbolic.

There are different proofs available for this fact. One can show for instance that the Dehn function of $D_{\lambda }$, $\lambda \in [-1,0)$, is exponential (the argument for $D_{-1}$ is outlined in [37]), and then use a result by Gromov [15] to deduce that $D_{\lambda }$, $\lambda \in [-1,0)$ is not Gromov-hyperbolic since the Dehn function is not linear. Another possibility would be to consider the asymptotic cone of $D_{\lambda }$, $\lambda \in [-1,0)$; see [4] and references therein. A proof for Proposition 3.1 is also contained in [9, §3.1], where it was observed that points in $D_{\lambda }$, $\lambda \in [-1,0)$, which are not contained in the same hyperbolic plane can be joined by quasi-geodesics that do not lie close to each other. We recall the argument below. It is convenient to think of the hyperbolic plane $\mathbb {H}^2$ not as the upper half plane $\{(u,v):\;v>0\}$ with the metric given by

$$\begin{aligned} \mathrm {d}s^2=\frac{1}{v^2}(\mathrm {d}u^2 + \mathrm {d}v^2), \end{aligned}$$

but rather to apply a coordinate transform $(x, {z})=(u,\log v)$. Then $\mathbb {H}^2$ can be seen as $\mathbb {R}^2$ equipped with the metric given by $\mathrm {d}s^2 = e^{-2z}\;\mathrm {d}x^2 + \mathrm {d}z^2$. It turns out that the groups $D_{\lambda }$, $\lambda \in [-1,0)$, are all foliated by isometrically embedded copies of $\mathbb {H}^2$. Perpendicular to these planes, there is another family of homothetically embedded ‘upside down’ versions of $\mathbb {H}^2$.

### Proof of Proposition 3.1 following [9]

Recall that $D_{\lambda }$ is $\mathbb {R}^3$ with the group law

$$\begin{aligned} (x,y,z)*(x',y',z')=(x+e^z x', y + e^{\lambda z}y',z+z'). \end{aligned}$$

Let $g_{\lambda }$ be the metric on $D_{\lambda }$ which makes the following left-invariant frame orthonormal:

$$\begin{aligned} E_1=e^z\partial _x,\quad E_2=e^{\lambda z}\partial _y,\quad E_3= -\partial _z. \end{aligned}$$

[Note that $\{E_1,E_2,E_3\}$ has structure constants as described in (1.2).] The associated length element is given by

$$\begin{aligned} \mathrm {d}s^2 = e^{-2z}\;\mathrm {d}x^2 + e^{-2 \lambda z}\;\mathrm {d}y^2 + \mathrm {d}z^2. \end{aligned}$$

It follows that the planes $\{y=\mathrm {const}\}$ are isometrically embedded copies of $\mathbb {H}^2$, whereas the planes $\{x=\mathrm {const}\}$ are homothetically embedded copies of the reflected hyperbolic plane.

Consider two points $p_1=(x_1,y_1,z_1)$ and $p_2=(x_2,y_2,z_2)$ in $D_{\lambda }$ with $x_1\ne x_2$ and $y_1\ne y_2$. We will construct two quasi-geodesics $\gamma _a$ and $\gamma _b$ which connect $p_1$ and $p_2$ but do not lie close to each other. First, we let $\gamma _{a,1}$ be the geodesic segment between $p_1$ and $(x_2,y_1,z_2)$ inside the hyperbolic plane $\{y=y_1\}$. Then we let $\gamma _{a,2}$ be the geodesic segment in $\{x=x_2\}$ connecting the endpoint of $\gamma _{a,1}$ to $p_2$, and we denote by $\gamma _a$ the concatenation of $\gamma _{a,1}$ and $\gamma _{a,2}$. The curve $\gamma _b$ is obtained in an analogous way, by first connecting $p_1$ to $(x_1,y_2,z_2)$ by a geodesic segment in the plane $\{x=x_1\}$, and then connecting the point $(x_1,y_2,z_2)$ to $p_2$ by a geodesic in the hyperbolic plane $\{y=y_2\}$. Observe that the map

$$\begin{aligned} D_{\lambda } \rightarrow \mathbb {H}^2 \times \mathbb {H}^2,\quad (x,y,z)\mapsto ((x,z),(y,z)) \end{aligned}$$

is a quasi-isometric embedding with constants depending only on the parameter $\lambda$ if $D_{\lambda }$ is endowed with the distance induced by $g_{\lambda }$ and $\mathbb {H}^2 \times \mathbb {H}^2$ is equipped with a product metric of $d_{\mathbb {H}^2}$, where $d_{\mathbb {H}^2}$ is induced by a metric of constant sectional curvature equal to $-1$. It follows that both $\gamma _a$ and $\gamma _b$ are (*L*, *C*)-quasi-geodesics, for constants $L=L(\lambda )\ge 1$ and $C=C(\lambda ) \ge 0$ independent of *a* and *b*. By applying this construction to a sequence of points $p_{1,n}=(x_{1,n},y_{1,n},z)$ and $p_{2,n}=(x_{2,n},y_{2,n},z)$, with $z\in \mathbb {R}$, $|x_{1,n}-x_{2,n}|\rightarrow \infty$ and $|y_{1,n}-y_{2,n}|\rightarrow \infty$ as $n\rightarrow \infty$, we see that there does not exist a constant $\delta >0$ such that for every *n*, the curve $\gamma _{a}$ connecting $p_{1,n}$ to $p_{2,n}$ is contained in the $\delta$-neighborhood of $\gamma _b$. This proves that $(D_{\lambda },g_{{\lambda }})$ is not Gromov hyperbolic. $\square$
